# Predictors of problems reported on the EQ-5D-3L dimensions among people with impaired vision in northern Portugal

**DOI:** 10.1186/s12955-022-02043-4

**Published:** 2022-09-06

**Authors:** Antonio Filipe Macedo, Amanda Hellström, Robert Massof, Hanna Tuvesson, Mikael Rask, Pedro Lima Ramos, Jalal Safipour, Ina Marteinsdottir, Evalill Nilsson, Cecilia Fagerström, Kristofer Årestedt

**Affiliations:** 1 Department of Medicine and Optometry, Faculty of Health and Life Sciences, Linnaeus University, 39182 Kalmar, Sweden; 2 Center of Physics, Optometry and Vision Science, University of Minho, Braga, Portugal; 3 Department of Health and Caring Sciences, Faculty of Health and Life Sciences, Linnaeus University, Kalmar, Sweden; 4 Wilmer Eye Institute, Johns Hopkins University School of Medicine, Baltimore, USA; 5 Department of Research, Region Kalmar County, Kalmar, Sweden

## Abstract

**Background:**

The EQ-5D index often fails to detect the effect of ophthalmic diseases and sight loss. Investigating predictors of individual EQ-5D health dimensions might reveal the underlying reasons. The aim of this study was to investigate predictors of health dimension ratings obtained with the EQ-5D-3L from participants with impaired vision representing a spectrum of eye diseases.

**Methods:**

Observational cross-sectional study with participants recruited at four public hospitals in Portugal. Outpatients with visual acuity of 0.30 logMAR(6/12) or worse in the better-seeing eye were invited to participate. Participants completed two instruments: the EQ-5D-3L (measures participants’ perceived health-related quality-of-life) and the Massof Activity Inventory (measures visual ability–ability to perform vision-related activities). This study used logistic regression models to identify factors associated with responses to the EQ-5D-3L.

**Results:**

The study included 492 participants, mean age 63.4 years (range = 18–93), 50% females. The most common diagnosis was diabetic retinopathy (37%). The mean visual acuity in the better seeing eye was 0.65 logMAR (SD = 0.48) and the mean visual ability was 0.62 logits (SD = 2.04), the correlation between the two was r = − 0.511 (p < 0.001). Mobility and self-care were the health dimensions with the fewest problems (1% reported extreme problems), anxiety and depression the dimension with the most problems (24% reported extreme problems). ROC curve analysis showed that the EQ-5D index was a poor predictor of cases of vision impairment whilst visual ability given was a good predictor of cases of vision impairment. Visual ability was an independent predictor of the response for all dimensions, higher ability was always associated with a reduced odds of reporting problems. The odds of reporting problems were increased for females in 3 out of 5 dimensions. Comorbidities, visual acuity and age-category were predictors of the odds of reporting problems for one dimension each.

**Conclusions:**

The odds of reporting problems for the five health dimensions of the EQ-5D-3L were strongly influenced by the ability to perform vision-related activities (visual ability). The EQ-5D index showed poor performance at detecting vision impairment. These findings are informative and relevant for the clinic and for research evaluating the impact of eye diseases and disease treatments in ophthalmology.

**Supplementary Information:**

The online version contains supplementary material available at 10.1186/s12955-022-02043-4.

## Introduction

It is estimated that about 3% of the European population over age 55 years suffers from disabling vision impairment [1]. Disabling vision impairment is typically defined as visual acuity in the better seeing eye worse than 0.5 logMAR (6/19) (logMAR corresponds to logarithm base 10 of the minimum angle of resolution) [1, 2] but acuity 0.3 logMAR (6/12) or worse is also considered to be a disabling vision impairment by, for example, the Centers for Disease Control and Prevention in the USA [3]. Eye diseases leading to uncorrectable visual impairments have been associated with an increased risk for deterioration in the quality of life. Even mild vision impairment is likely to interfere negatively with the performance in vision-related activities, particularly in domains such as reading or mobility [4–7]. Previous studies have used patient‐reported outcome measures to assess health-related quality of life (HRQoL) in patients with eye diseases, that included condition-specific measures to address vision-specific domains e.g., the 25-item National Eye Institute Visual Function Questionnaire [8], and generic measures to address more general domains of HRQoL, e.g., the EQ-5D or the WHO Quality of Life 100 (WHOQOL-100) [4, 5, 7, 9–11].

Previous research has highlighted some of the main factors contributing to a deterioration of HRQoL in patients with eye diseases. The most commonly reported factors are reduced visual acuity [5, 6, 9], vision-related disability [5, 12] and other comorbid chronic conditions such as diabetes, stroke, and rheumatoid arthritis [9, 13]. Among these factors, reduced visual acuity is considered the main factor responsible for poor HRQoL in patients with chronic eye diseases due to the direct and negative impact of vision impairment on patients’ functioning [5, 9, 14, 15].

The EQ-5D-3L is commonly used to measure the burden of vision loss and the burden of specific eye diseases [16–18]. The EQ-5D-3L is based on five health dimensions that can be used to describe the patient’s health state from their own perspective, the dimensions are: mobility, self-care, usual activities, pain/discomfort, and anxiety/depression. In addition, the EQ-5D-3L includes a visual analogue scale (EQ VAS), that reflects individuals’ overall current health. Valuations of health states generated by the EQ-5D-3L have been made for the general public in many countries, including Portugal [19]. The scoring system to compute the EQ-5D index is based on preferences; that is, the problems on each dimension are weighted to reflect how good or bad people think they are. Some studies have shown that problems with pain and discomfort often carry more weight than problems with self-care as reported by the EQ-5D, and this is reflected in the way questionnaire respondents’ profile data is summed to produce the EQ-5D index [20, 21].

The EQ-5D index is the most commonly used indicator to compute quality-adjusted life years (QALY). QALYs represent the benefit of a health intervention in terms of time in a series of quality-weighted health states, in which the quality weights reflect the desirability of living in the state, typically from “perfect” health (weighted 1.0) to dead (weighted 0.0) [22]. QALYs are fundamental to, for example, cost-effectiveness studies [18, 22–26]. Some studies have shown that the EQ-5D index may fail to capture the impact of ophthalmological interventions [16, 17, 27–31] and similar issues have been raised, for example, in mental health interventions [32–34]. Nevertheless, the EQ-5D remains the most commonly used generic measure of HRQoL in studies assessing the impact of vision disorders [35].

Some studies have sought to map the EQ-5D index from vision-related quality of life measures, such as the 25-item National Eye Institute Visual Function Questionnaire, but the mapping was poor [8, 36], That is, the best equation to convert values from one instrument to the other exhibited poor fitting to the data. The authors pointed out that the EQ-5D index was poor at discriminating between levels of ability to perform vision-related activities [36, 37], in other words, the EQ-5D index was poor at discriminating visual ability. Using an instrument such as the EQ-5D-3L remains convenient to compute QALYs across diseases or interventions. For example, this allows one to compare an intervention that takes a visually impaired person from a value of 0.1 to 0.3 with an intervention that takes a healthy person with back pain from 0.8 to 1.0 [22]. However, as mentioned, the EQ-5D index is anchored on measures of perfect health and death and, therefore, is an indirect measure of the real scores given to each dimension. That is, the index is a preference-based measure and does not reflect the respondent’s own perceptions.

Previous studies failed to investigate what would be the predictors for different health dimensions and knowledge is lacking in this area. Further understanding of the underlying issues with the EQ-5D index in ophthalmic diseases can be gained by investigating predictors of individual EQ-5D health dimensions. This is of particular importance at a time when new treatments such as gene therapy are becoming available and cost-effectiveness studies are needed [38, 39]. The aim of this study was to identify predictors of health dimensions measured by the EQ-5D-3L in a sample of people with impaired vision due to a spectrum of eye diseases.

## Methods

### Design and participants

This observational study was based on cross-sectional data. Participants were recruited from four public hospitals with ophthalmology departments in northern Portugal between July 2014 and January 2016. Outpatients at the departments with the latest recorded visual acuity of 0.30 logMAR (6/12) or worse in the better seeing eye were invited to take part in the study [40]. Approximately 3000 patients were invited by letter by their hospital. The letter included information about the study and a consent form. The precise number of letters delivered is unknown because some were returned to the sender or lost in the mail system. A total of 546 patients agreed to participate by returning a signed consent form to the research group. Patients, henceforth called “participants”, were contacted by phone by a research assistant and a research visit at the hospital was scheduled. Fifty-four participants were excluded due to, for example, incomplete data (e.g., failure to respond to both questionnaires) or because they were younger than 18 years.

### Data collection

Sociodemographic and clinical characteristics of the participants were retrieved from the medical records. Visual acuity was measured as part of the research visit using a back-illuminated Early Treatment Diabetic Retinopathy Study chart (ETDRS, luminance 85 cd/m^2^) [41] at 4, 2 or 1 m according to the severity of vision loss. The room lights were extinguished during measurements. Visual acuity is defined in this study as the limit of visual resolution in logMAR units estimated from the number of correctly read letters on the chart, each letter corresponds to 0.02 logMAR units when using letter-by-letter scoring [41, 42]. In this study, a completely blind eye without light perception was classified as having acuity 2.7 logMAR. Acuity of 1.02 logMAR or worse was considered severe vision impairment, normal visual acuity fluctuates between − 0.3 and 0.0 logMAR. Comorbidities were classified in one of the 16 categories given in Table 1, comorbidities were both retrieved from the medical records and self-reported (asked) during the research visit.

**Table 1:** Summary of the sociodemographic and clinical characteristics of the participants in the current study (n = 492)

*Age*	*Mean (SD)*	*Diagnosis*	% (*n*)
	63.4 (14.2) years	Diabetic Retinopathy	37 (182)
*Acuity*		Age-Related Macular Degeneration	13 (63)
	0.65 (0.48) logMAR	Glaucoma	10 (51)
*Sex*	% (*n*)	Disorders of the Globe	8 (41)
Females	50 (246)	Corneal Disorders	8 (37)
Males	50 (246)	Other Retinal Disorders	6 (28)
*Age category*	% (*n*)	Unknown	4 (19)
65 or less	51 (248)	Cataract	3 (17)
More than 65	49 (244)	Retinal Detachments and other defects	3 (16)
*Education*	% (*n*)	Optic Nerve Disorders	3 (15)
Less 4 years	10 (48)	Disorders of the Choroid	3 (14)
4 years	52 (254)	Other Eye Disorders	2 (9)
6 years	14 (71)		
9 years	12 (57)	*Household income per person*	% (*n*)
12 years	7 (33)	Less than 485 euro	45 (220)
Degree	4 (18)	Between 485 and 1000 euro	40 (197)
Unknown	2 (11)	More than 1000 euro	13 (63)
*Marital status*	% (*n*)	Unknown	2 (12)
Married	65 (320)		
Widowed	15 (76)	*Comorbidities*	% (*n*)
Single	13 (63)	Allergies	10 (47)
Divorced	6 (30)	Stroke	9 (42)
Other	0 (2)*	Cancer	4 (20)
*Household living arrangements*	% (*n*)	Diabetes	46 (226)
w/ spouse and children	64 (313)	Autoimmune	2 (9)
w/ children	16 (80)	Heart condition	16 (79)
alone	12 (58)	Endocrine condition	4 (21)
w/ relatives	5 (23)	Gastrointestinal condition	12 (60)
w/ spouse	3 (14)	Liver disease	2 (9)
Other	1 (4)	Musculoskeletal disorder	26 (130)
*Employment status*	% (*n*)	Pulmonary disease	6 (30)
Retired	52 (255)	Thyroid condition	7 (34)
Early retired	20 (98)	Hypertension	37 (184)
Working	11 (54)	Hearing impairments	10 (47)
Unemployed	10 (49)	Neurologic problems	3 (14)
Other	6 (29)	Psychological problems	12 (57)
Sick leave	1 (7)		

w/, corresponds to “with”; *percentage is 0 because decimals have been removed

### The questionnaires

The questionnaires were the EQ-5D-3L (three level version of the EQ-5D) and the Mass of Activity Inventory (MAI). Both questionnaires were completed at the hospital during the research visit, questions were read aloud because most participants were unable to read printed text.

The EQ-5D-3L is a generic instrument that is used to classify the participants’ perceived HRQoL. The instrument consists of five health dimensions: mobility, self-care, usual activities, pain/discomfort and anxiety/depression. Each dimension is rated on a three-point scale; ‘no problems’ (1), ‘some problems’ (2), and ‘extreme problems’ (3). The dimensions can be reported separately as health states (for example 11111 corresponds to “no problems” in all dimensions) or as a unique descriptive health profile—that is, the EQ-5D index [20, 43]. Valuations of health states generated by the EQ-5D-3L used in the current study have been made for the general public in Portugal [19]. The values, or utilities, are set on a scale on which 0 corresponds to death and 1 to a state of perfect health (EQ-5D index), a negative value represents a state “worse than death.”

The MAI is an adaptative questionnaire designed to provide an individualized assessment of difficulties of a visually impaired respondent when performing valued activities. Disabilities, or activity limitations according to the World Health Organization’s International Classification of Functioning, occur when an individual reports abnormal difficulty in achieving important goals. Difficulties achieving a goal are said to depend on the difficulty experienced in the tasks that underlie each goal. The MAI consists of a hierarchal structure in which specific cognitive and motor vision-dependent tasks (e.g., pouring or mixing without spilling) underlie more global goals (e.g., preparing meals). The instrument can be used to measure the overall visual ability and visual ability in 4 functional domains: reading, mobility, visual motor function, and visual information processing [44–46]. We used the Portuguese version of the MAI that we translated and tested psychometrically [47]. In the Portuguese version of the MAI, participants were questioned about difficulties with 46 goals. Goals rated “not important” are skipped, for goals rated “slightly” to “very important” respondents were asked to rate the goal’s difficulty on a five-point scale ranging from “not difficult” = 4 to “impossible to do” = 0. The difficulty responses were Rasch analysed with the Andrich rating scale model [48] and Winsteps program (v4.0) to produce a continuous measure of “visual ability” given by the output variable “person measure”. Calibration values for the items were gently provided by Goldstein and colleagues [46, 49]. Fitting data to the Rasch model places item and person parameter estimates on the same log-odds units (logit) scale, which gives a linear transformation of the raw score. Our numeric coding of the scale dictates that higher person measures correspond to better visual ability.

### Statistical analysis

Descriptive statistics was used to provide information about the sociodemographic and clinical characteristics of the participants. Pearson correlations between visual acuity, ability and EQ-5D index were tested. Logistic regression models were used to identify factors associated with the health dimensions in the EQ-5D-3L. Multiple ordinal logistic regression, i.e., partial proportional odds regression models, were used for three of the EQ-5D health dimensions: usual activities, pain and discomfort and anxiety and depression. For mobility and self-care, levels 2 and 3 were merged due to a small number of responses with “extreme” problems. Therefore, these two health dimensions were analysed with binary logistic regression.

The initial predictors for all models are given in Additional file 1: S1. Age was dichotomized using a cut-off at 65 years because that is the typical age for pension in Portugal and we assumed that people of working age or retirement age could have different perspectives. We expected a low but relevant contribution to our answers from acuity in the worse seeing eye; therefore, we also dichotomized this predictor using the cut-off acuity of severe vision impairment (1.02 logMAR) [50]. The assumption is that people with severe vision impairment in the worse seeing eye will experience more reduced binocular visual field which can impact their perceived difficulties.

The multiple partial proportional odds regression models were fitted with PROC LOGISTIC in SAS software (SAS Institute Inc., Cary, NC, USA). According to the available documentation from the SAS Institute the model was set to allow automatic selection of equal slopes or unequal slopes for each predictor [51, 52], statistical significance was set at p < 0.05 and only main effects were tested. An example of the SAS software code used is provided in Additional file 1: S2.

ROC (receiver operating characteristic) curves were used to determine the area under the curve (AUC) to assess sensitivity and the specificity on the MAI (visual ability in logits) and the EQ-5D index to predict vision impairment. A true positive case of impairment (test result 1) was defined when acuity in the better seeing eye was worse than 0.5 logMAR.

## Results

### Characteristics of the participants

The final sample for the current study included 492 participants. Table 1 summarizes their sociodemographic and clinical characteristics. The mean age was 63.4 years (range = 18–93) and equal numbers of males and females were included, 246 respectively. In total, 27% of the participants reported no comorbidities, 73% reported one or more comorbidities and diabetes was the most frequently reported comorbidity (46%). The most common cause of vision impairment was diabetic retinopathy (n = 182 or 37%).

The mean visual acuity in the better eye was 0.65 logMAR (SD = 0.48), median 0.50 logMAR (IQR = 0.48). The mean visual ability was 0.62 logits (SD = 2.04) and the median visual ability was 0.34 logits (IQR = 2.50). The mean EQ-5D index was 0.53 (SD = 0.29) and the median was 0.55 (IRQ = 0.40).

**Fig. 1:**
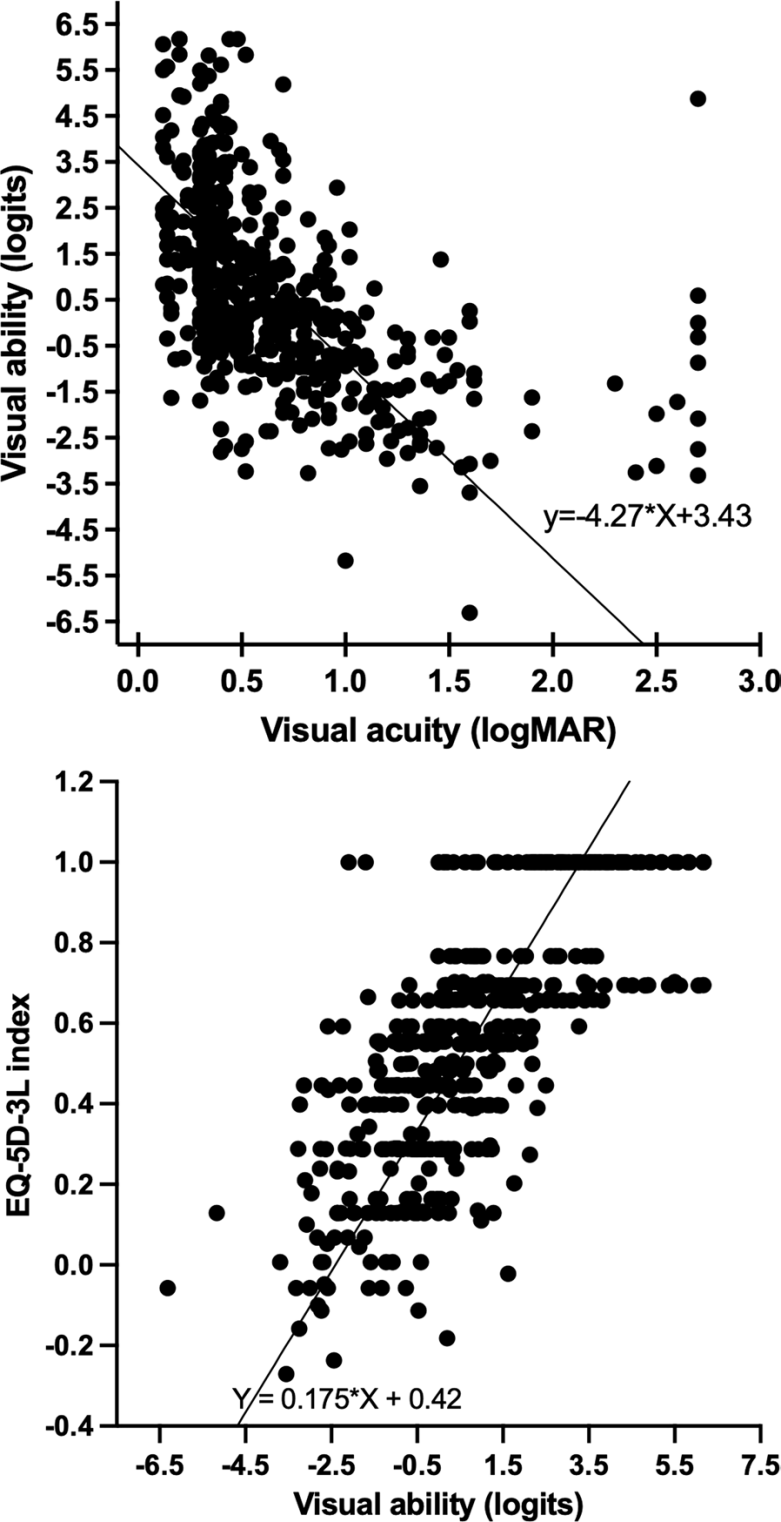
Left graph -scatter plot showing the variation in visual ability with visual acuity in the better seeing eye. Right graph-scatter plot showing the variation in EQ-5D index with visual ability. Lines show fitting using Deming regression fitted with R-package [53]

**Fig. 2:**
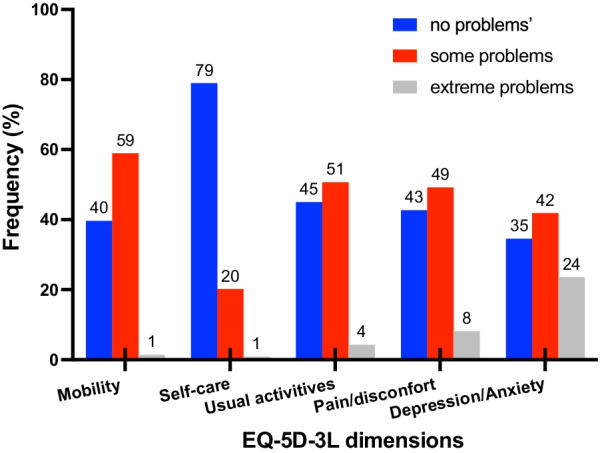
Summary of the answers to each health dimension of the EQ-5D. Numbers at the top of the columns indicate the prevalence of problems (percentage)

Figure 1 shows the correlation between acuity and ability (r = − 0.511, p < 0.001) and the correlation between ability and the EQ-5D index (r = 0.729, p < 0.001). Figure 2 shows the prevalence of problems in each of the 5 dimensions of the EQ-5D-3L.

**Fig. 3:**
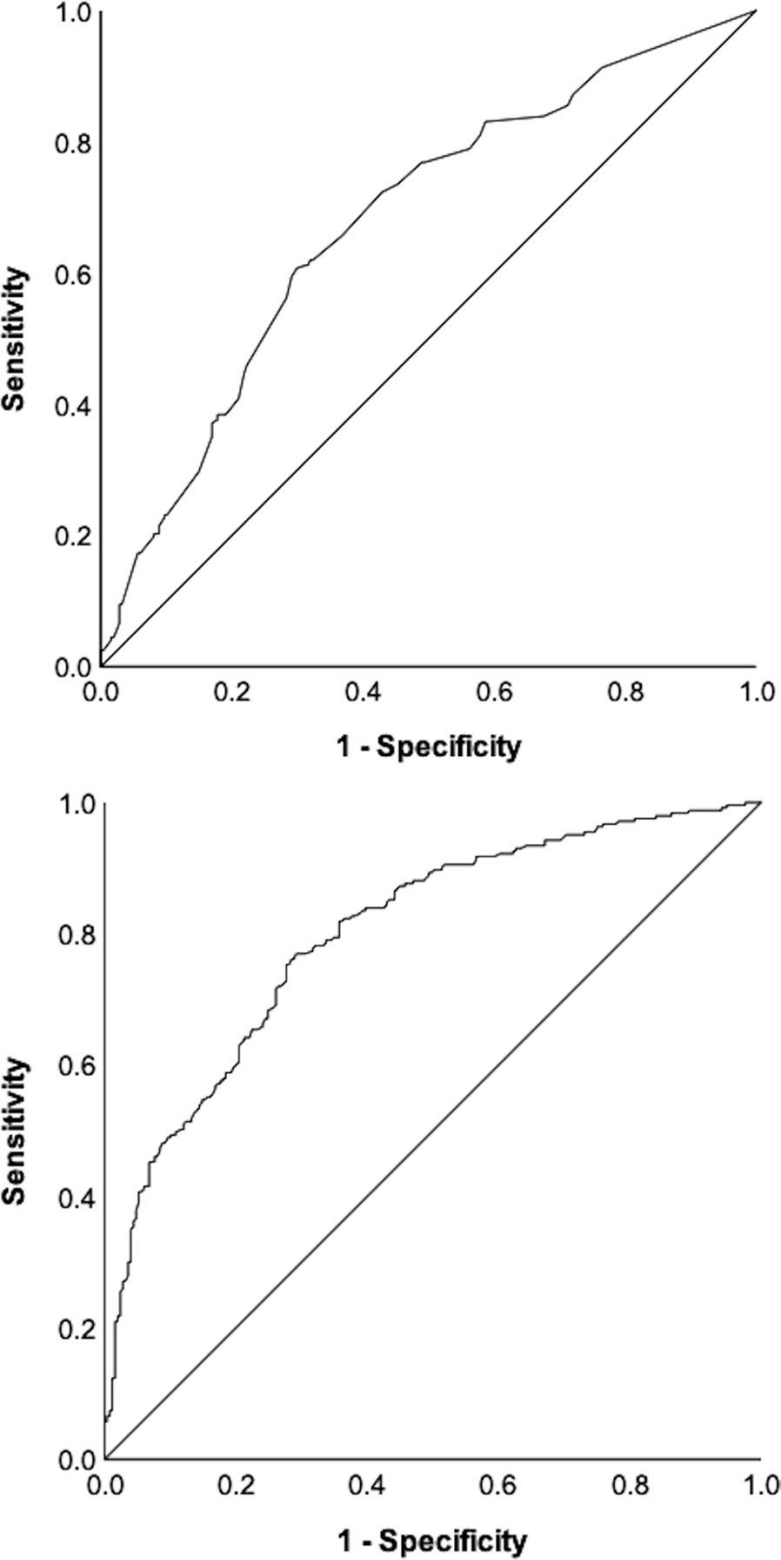
ROC curves showing the sensitivity and the specificity of the EQ-5D-3L (Top chart) and the MAI (Right chart) to detect cases of vision impairment defined as visual acuity in the better eye worse than 0.5 logMAR

Graphs with the ROC curves to determine the ability of the questionnaires to detect cases of vision impairment (acuity in the better seeing eye worse that 0.5 logMAR) are given in Fig. 3. For the EQ-5D-3L the AUC was 0.679 (95%CI: 0.630–725, p < 0.001) and for MAI the AUC was 0.801 (95%CI: 0.762–839, p < 0.001). With a cut-off of 0.385 logits for the MAI, the sensitivity was 0.749 and specificity was 0.723. With a cut-off of 0.619 for the EQ-5D-3L, the sensitivity was 0.769 and specificity was 0.510. These results show that the EQ-5D index was a poor predictor of cases of *vision impairment* whilst visual ability given by the MAI was a good predictor of cases of *vision impairment*. In Additional file 1: S4 we also provide the AUC for cases of *severe vision impairment* (acuity in the better seeing eye worse that 1.0 logMAR), for this scenario the AUC for both questionnaires improved (0.740 for the EQ-5D-3L and 0.860 for the MAI).

### Multiple logistic regression models

The results of the final logistic regression models are summarized in Table 2, only significant predictors using a stepwise selection are reported (see Additional file 1: S2 for model specification using stepwise selection). Models attempt to predict scores “moderate” and “extreme” problems. Due to the low number of participants that endorsed the response option “extreme problems” for mobility and self-care this alternative was collapsed with the response option “moderate problems” for these two items (Fig. 2). The results in Table 2 follow a pattern in which the odds of reporting moderate or severe problems always decrease when visual ability (given by the MAI) increases. Being female increased the odds of reporting moderate or extreme problems in 3 out of 5 dimensions. Age-category “65 or less years” increased the odds and acuity (continuous variable) reduced the odds of reporting problems in pain and discomfort. Higher acuity reduced the odds of reporting problems with pain and discomfort (higher acuity values correspond to worse vision). Having no comorbidities increased the odds of reporting problems with usual activities.

**Table 2:** Summary of the logistic regression models for each dimension with significant predictors. In all models the reference category was “no problems”

Dimension	Predictor	Category	Odds Ratio	95% CI	p-value
Mobility*					
“moderate” or	Sex	Females	1.77	1.10–2.83	0.018
“extreme”	Visual ability	–	0.37	0.31–0.44	< 0.001
Self-care*					
“moderate” or “extreme”	Visual ability	–	0.37	0.30–0.46	< 0.001
*Usual activities*					
“moderate”	Comorbidities	No comorbidities	2.29	1.38–3.79	< 0.001
	Visual ability		0.33	0.27–0.40	0.001
“extreme”	Comorbidities	No comorbidities	2.29	1.38–3.79	0.001
	Visual ability		0.23	0.14–0.38	< 0.001
*Pain/discomfort*					
“moderate”	Age-Category	65 or less years	1.56	1.08–2.30	0.013
	Sex	Females	1.99	1.37–2.87	< 0.001
	Acuity	–	0.55	0.35–0.87	0.025
	Visual ability	–	0.62	0.55–0.69	0.001
“extreme”	Age-Category	65 or less years	1.56	1.08–2.30	0.013
	Sex	Females	1.99	1.37–2.87	< 0.001
	Acuity	–	0.55	0.35–0.87	0.025
	Visual ability	–	0.62	0.55–0.69	0.001
*Anxiety/depression*					
“moderate”	Visual ability	–	0.50	0.47–0.56	< 0.001
“extreme”	Sex	Females	2.68	1.65–4.37	< 0.001
	Visual ability	–	0.50	0.47–0.56	< 0.001

*Owing to small number of responses with ‘‘extreme problems”, respondents with “moderate” and “extreme’’ were combined to calculate odds ratio for ‘‘problems’’. When the proportional odds assumption for scores was valid the odds ratio is the same for moderate and extreme responses as is, for example, the case for all predictors in Pain/Discomfort. See Additional file 1: S2 and S3 for further clarifications

### Discussion

In this study we investigated predictors of the EQ-5D-3L dimensions in participants with impaired vision caused by a spectrum of eye diseases. The dimension in which problems (moderate or severe) were more prevalent was anxiety/depression and the dimension in which problems were less prevalent was self-care. These findings are in line with previous studies, but the prevalence of problems seems higher in our sample [19, 54]. Visual ability measured by the MAI assesses the perceived activity limitations imposed by impaired vision. Higher values of visual ability were consistently associated with lower odds of reporting problems in all dimensions of the EQ-5D-3L. These results are confirmed by the strong association between ability and the EQ-5D index. Sex, comorbidities, acuity and age-category were also identified as significant predictors for some health dimensions. It must be noted that visual ability was associated with visual acuity, meaning that the effect of reduced vision is somewhat captured by the activity limitations reported in the MAI.

The ROC curve analysis showed that the EQ-5D index was a poor predictor of cases of vision impairment and its predictive ability improved for cases of severe vision impairment. The problem with the EQ-5D index may be the scoring system when problems on each dimension are weighted to reflect how good or bad people think they are. Previous studies have shown that problems with pain and discomfort carry more weight than problems with self-care and this is reflected in the way questionnaire respondents’ profile data is summed to produce the EQ-5D index [20, 21]. For people with impaired vision pain and discomfort is likely to carry less weight than problems with self-care. That is, the way that health dimensions have been weighted during the valuation of health states for the general population may be the cause of the problems with the EQ-5D index in people with ophthalmic diseases and vision loss.

The odds of reporting problems were associated with reported activity limitations imposed by vision impairment. These results show that eye diseases or interventions that change substantially the ability to perform tasks that rely on vision are expected to have an impact on health dimensions; conversely, when eye diseases or interventions fail to change perceived activity limitations, they are unlikely to lead to significant changes in the health dimensions of the EQ-5D-3L. These results are important because they give further understanding of why the EQ-5D index, whose computation is based on scores for each dimension, has been considered “unresponsive” to the effect of sight-threating eye diseases such as diabetic retinopathy, diabetic macular oedema [55], primary open-angle glaucoma [31] or even, in some instances, to interventions such as cataract surgery [17, 56]. The EQ-5D-3L showed inconsistent results in conditions such as conjunctivitis [57] or endophthalmitis [58]. These eye conditions are unlikely to cause activity limitations. In the case of conjunctivitis only pain was significantly worse in patients with seasonal allergic conjunctivitis than in a control group. Although not statistically significant, in some cases the remaining domains were worse in the control group [57]. It is possible that in these studies visual ability was mostly unchanged by the eye condition or by the intervention and, because of that, changes in health dimensions and in the EQ-5D-3L index were deemed non-significant. At this stage it is important to discuss possible reasons for the link between visual ability and the reported problems in different dimensions.

Our findings show that the first 3 dimensions: mobility, self-care and usual activities-were more strongly modulated by visual ability (odds ratio below 0.5) than the remaining two dimensions (odds ratio 0.5 or more). These findings are consistent with previous studies showing that people with eye diseases are more likely to report problems in the first 3 dimensions [29, 31, 54, 59]. The link between visual ability and the first 3 dimensions is intuitive because these, like visual ability, are expected to assess activity limitations. However, visual ability was also a predictor for pain/discomfort and for depression/anxiety. Recent studies have shown that depression/anxiety are common in people with eye diseases [60–62]. In the case of depression, it is possible that there is a bidirectional relationship with visual ability. In one way depression can reduce the motivation to perform tasks which can lead to perceived reduction of visual ability; conversely, a reduced ability to perform tasks might increase the risk of depression. Most diagnoses given in Table 1 are unlikely to cause significant pain per se; however, people diagnosed with retinal or corneal diseases are likely to report, for example, severe photophobia that causes pain [63] and difficulties to read that causes discomfort [64]. Therefore, reduced odds of problems with pain and discomfort are expected in people who continue to perform activities (better visual ability). Nevertheless, people continue to perform activities because they are, eventually, less prone to pain and discomfort when performing them. Given this explanation, as discussed for depression, it is likely that the relationship between visual ability and pain and discomfort is bidirectional.

Other predictors of reporting moderate or severe problems in the current sample included: female sex, comorbidities, younger age and acuity. The odds were higher for females than for males in mobility, pain/discomfort, and anxiety/depression. These results are in line with other studies in the general population [65] or in clinical samples in which females more often report problems in most dimensions [54, 59, 66, 67]. These results add to previous evidence that it is important to consider sex as a possible confounder when designing studies with the EQ-5D-3L. Having no comorbidities increased the odds of reporting problems with usual activities. We speculate that this happens because participants with no comorbidities are more likely to link their problems with usual activities to vision than people suffering from other comorbidities. Higher values of acuity reduced the odds of reporting problems in pain and discomfort. It must be noted that higher values of acuity correspond to more impaired vision and one unit of acuity is a large step: acuity 0.00 logMAR corresponds to “normal vision” and 1.02 to “severe vision impairment”. A possible explanation for this result is that people with more impaired vision are more likely to have a stable disease and receive less invasive treatments. In line with that, the increased odds of reporting pain and discomfort among participants 65 years or less might be due to early stages of the disease and the number of treatments, often invasive injections into the eye, that they must receive [68].

This study has some limitations. One limitation was the inability to use the EQ VAS because many participants were unable to see it. When we tried to use the EQ VAS by suggesting “pick a number between 0 and 100” participants were confused and often we were forced to start to give suggestions. An additional limitation was our inability to compare the profile of the participants with the profile of those who were invited because some invitation letters were lost. Our estimated 18% response-rate for the research interviews can be considered low; although, this is a conservative low estimation because not all patients received the letter. Non-participants were asked by telephone if they could tell the main reason for refusing to participate, the most commonly reported reasons were: “I am too debilitated to participate”, “It is far away from my home”, “There are no benefits in participating”, and “I have no one to go with me” [40]. This was a cross-sectional study and, therefore, no causal conclusions can be drawn.

In conclusion, the results of the current study show that responses to the dimensions of the EQ-5D-3L are strongly influenced of by visual ability, that is, the ability to perform activities that rely on vision and are relevant for the individual. The results of this investigation should be considered when planning and evaluating interventions in ophthalmology in which the EQ-5D-3L (or more modern version of the instrument) and QALYs are expected to be used as relevant outcome measures.

## Supplementary Information

**Additional file 1.** Supporting additional information for methods and results.

## Data Availability

The datasets analysed during the current study are available from the corresponding author on reasonable request.
