# Supplementary material for: Predictors of problems reported on the EQ-5D-3L dimensions among people with impaired vision in northern Portugal
Source: Health Qual Life Outcomes. 2022 Sep 6;20:132. doi: 10.1186/s12955-022-02043-4 (PMC9450368; doi:10.1186/s12955-022-02043-4)
Supplement: Supplementary file 1 — Additional file 1. Supporting additional information for methods and results. [file 12955_2022_2043_MOESM1_ESM.docx]

# Title

# Manuscript title: Predictors of problems reported on the EQ-5D-3L dimensions among people with impaired vision in northern Portugal

# Supplemental materials

## S1-Summary of the predictor variables included in the logistic model (table) and list of comorbidities

| **Predictor** | **levels** | **value** |
| --- | --- | --- |
| **Sex** | male | 0 |
|  | female | 1 |
| **Age-Category**** | 65 years or less | 0 |
|  | >65 | 1 |
| **Comorbidities** | no-comorbidities | 0 |
|  | 1 or more | 1 |
| **Visual acuity in the worse seeing eye*** | visual acuity 1.02 or worse | 0 |
| **Defined as “Acuity-Category”** | visual acuity 1.0 or better | 1 |
| **Visual acuity in the better seeing eye**  **Defined “Acuity”** | continuous | - |
| **Visual ability (defined as AI)** | continuous | - |
| **Higher values correspond to worse acuity, minimum step on the scale is 0.02 logMAR* | | |

## S2-The image below shows an example of code used to predict scores given to Usual activities (eq5d3 in the code)


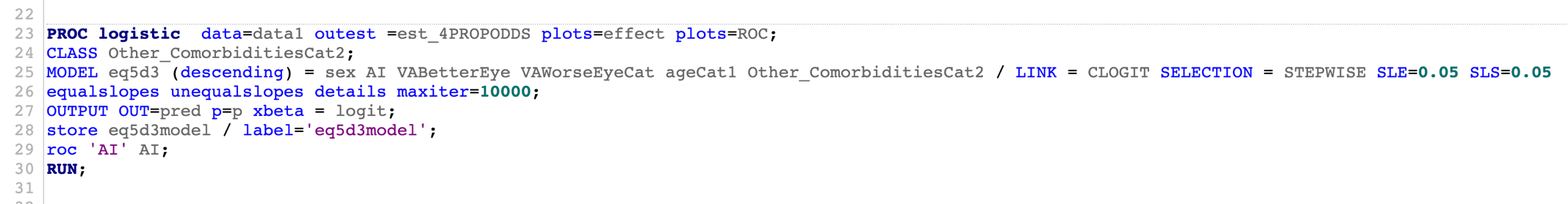


*With the model specified as shown above (line 26) all predictors in both their equal and non-equal (general) slope forms across the response levels are available for selection. These predictors are then simultaneously evaluated in a stepwise manner to build the model.*

In the MODEL:

- sex: male = 0, female =1
- AI =visual ability given by the Activity Inventory (continuous)
- VABetterEye: visual acuity in the better seeing eye (continuous)
- VAWorseEyeCat (visual acuity in the worse seeing eye): visual acuity 1.02 or worse (higher values correspond to worse acuity) = 0, visual acuity 1.0 or better = 1
- ageCat1: 65 years or less = 0, >65 = 1
- Other_ComorbiditiesCat2: YES = 0, NO = 1

## S3-Comparison of the empirical logits for the different predictors

| **The graphs below show that the slope of the lines with “estimated empirical logits” is not the same for “Usual activities” but is similar for “Pain and discomfort”,** |
| --- |
| **Usual activities** |
|  |
| 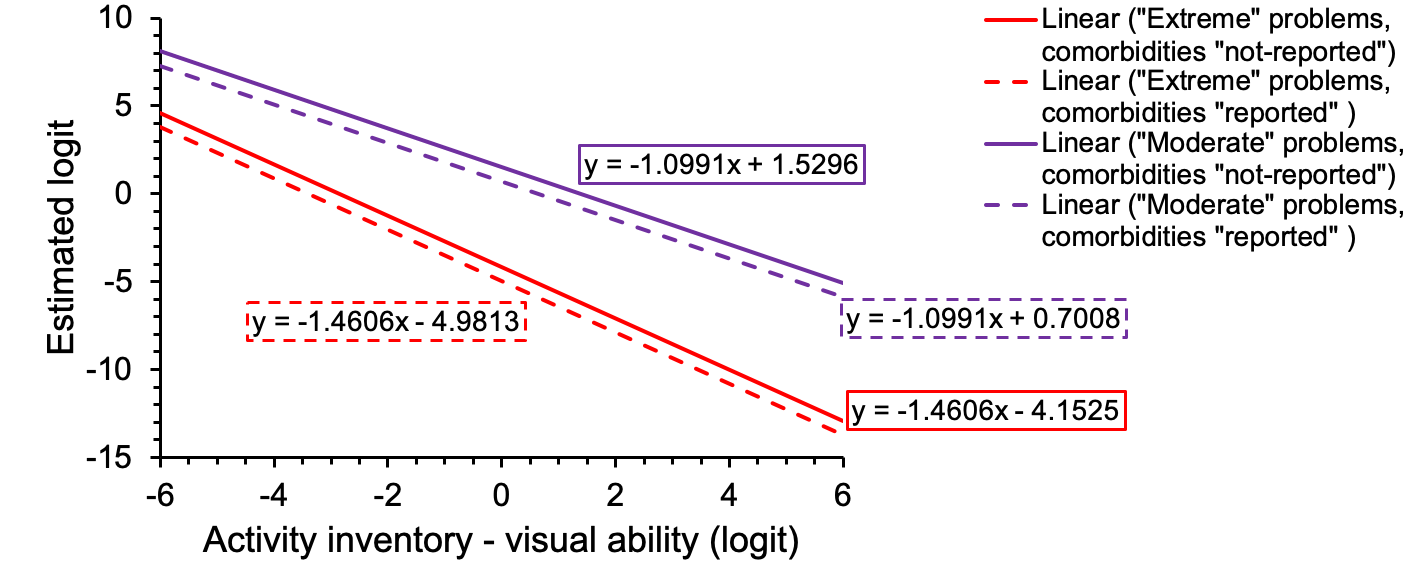 |
| *Figure legend: changes in estimated logit (y-axis), associated with an increase of the independent variable visual ability given by the activity inventory (x-axis). The slope for the lines was different for different scores of the EQ-5D-3L, the 2 top lines represent “moderate” problems, the 2 lines at the bottom correspond to “extreme” problems. The gap between the lines in both cases reflects the effect of comorbidities.* |
|  |
| **Pain and discomfort** |
|  |
| 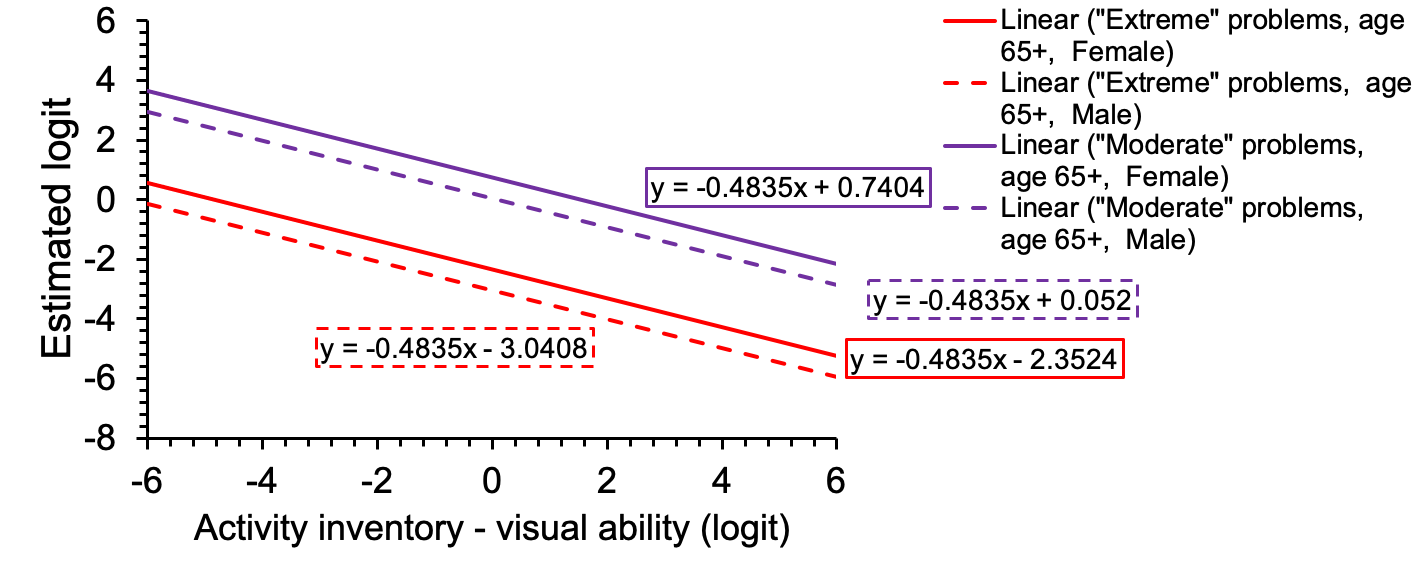 |
| *Figure legend:* ***c****hanges in estimated logit (y-axis), associated with an increase of the independent variable visual ability given by the activity inventory (x-axis). The slope for the lines was the same for different scores of the EQ-5D-3L,the 2 top lines represent “moderate” problems, the 2 lines at the bottom correspond to “extreme” problems. The gap between the lines in both cases reflects the effect of sex. For this representation visual acuity was set at its mean value for the sample, that was 0.656 logMAR.* |

## S4-ROC curves

## ROC curves showing the sensitivity and the specificity of the EQ-5D-3L and the MAI to detect cases of severe vision impairment defined as visual acuity in the better eye worse than 1.0 logMAR

| 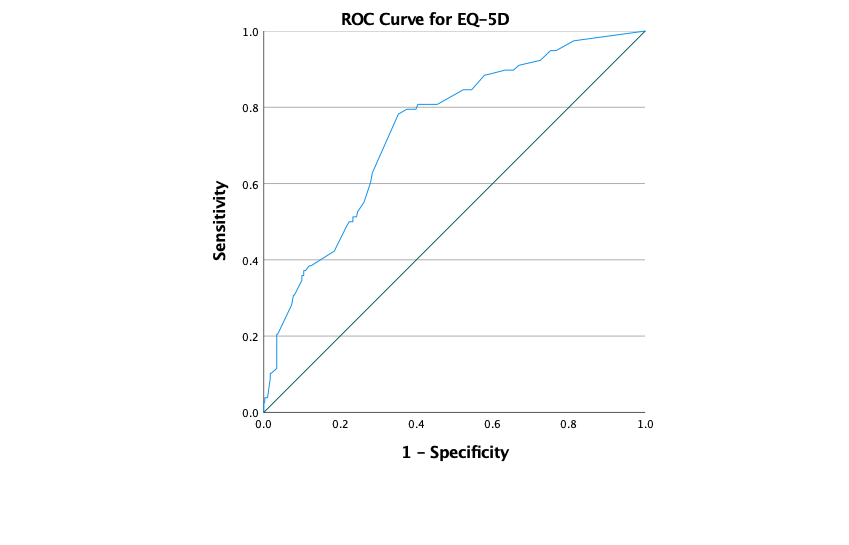 | 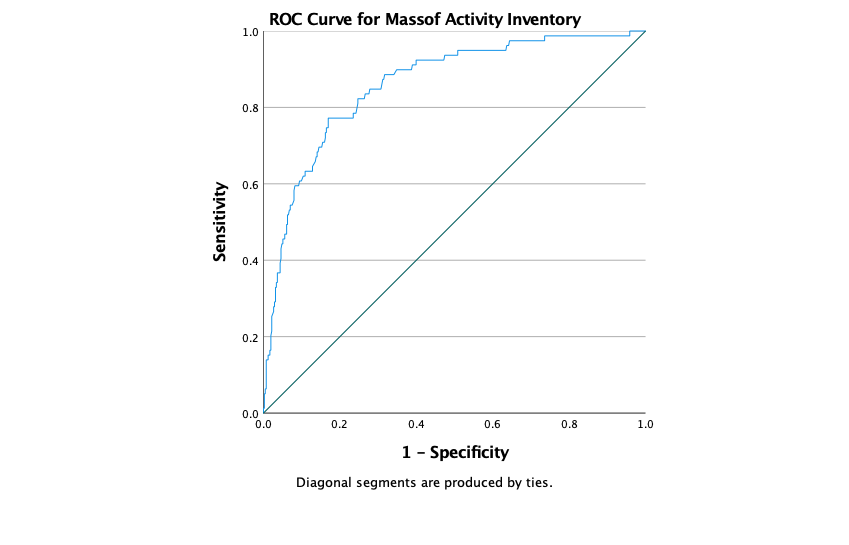 |
| --- | --- |
| 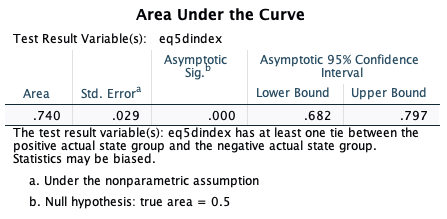 | 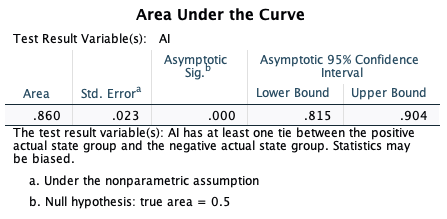 |
